# The Major Antigenic Membrane Protein of “*Candidatus* Phytoplasma asteris” Selectively Interacts with ATP Synthase and Actin of Leafhopper Vectors

**DOI:** 10.1371/journal.pone.0022571

**Published:** 2011-07-25

**Authors:** Luciana Galetto, Domenico Bosco, Raffaella Balestrini, Andrea Genre, Jacqueline Fletcher, Cristina Marzachì

**Affiliations:** 1 Istituto di Virologia Vegetale, Consiglio Nazionale delle Ricerche, Torino, Italy; 2 Dipartimento di Valorizzazione e Protezione delle Risorse Agroalimentari - Entomologia e Zoologia Applicate all'Ambiente, Università degli Studi di Torino, Grugliasco, Italy; 3 Istituto di Protezione delle Piante, Consiglio Nazionale delle Ricerche, Torino, Italy; 4 Dipartimento di Biologia Vegetale, Università degli Studi di Torino, Torino, Italy; 5 Entomology and Plant Pathology Department, Oklahoma State University, Stillwater, Oklahoma, United States of America; Indian Institute of Science, India

## Abstract

Phytoplasmas, uncultivable phloem-limited phytopathogenic wall-less bacteria, represent a major threat to agriculture worldwide. They are transmitted in a persistent, propagative manner by phloem-sucking Hemipteran insects. Phytoplasma membrane proteins are in direct contact with hosts and are presumably involved in determining vector specificity. Such a role has been proposed for phytoplasma transmembrane proteins encoded by circular extrachromosomal elements, at least one of which is a plasmid. Little is known about the interactions between major phytoplasma antigenic membrane protein (Amp) and insect vector proteins. The aims of our work were to identify vector proteins interacting with Amp and to investigate their role in transmission specificity. In controlled transmission experiments, four Hemipteran species were identified as vectors of “*Candidatus* Phytoplasma asteris”, the chrysanthemum yellows phytoplasmas (CYP) strain, and three others as non-vectors. Interactions between a labelled (recombinant) CYP Amp and insect proteins were analysed by far Western blots and affinity chromatography. Amp interacted specifically with a few proteins from vector species only. Among Amp-binding vector proteins, actin and both the α and β subunits of ATP synthase were identified by mass spectrometry and Western blots. Immunofluorescence confocal microscopy and Western blots of plasma membrane and mitochondrial fractions confirmed the localisation of ATP synthase, generally known as a mitochondrial protein, in plasma membranes of midgut and salivary gland cells in the vector *Euscelidius variegatus.* The vector-specific interaction between phytoplasma Amp and insect ATP synthase is demonstrated for the first time, and this work also supports the hypothesis that host actin is involved in the internalization and intracellular motility of phytoplasmas within their vectors. Phytoplasma Amp is hypothesized to play a crucial role in insect transmission specificity.

## Introduction

Phytoplasmas, wall-less plant pathogenic bacteria of the Class Mollicutes, infect a wide variety of herbaceous and woody plants, causing significant economic losses on cultivated crops worldwide [1]. Because they have yet to be cultured axenically, phytoplasmas are described as “*Candidatus* Phytoplasma spp.” [2]. Phytoplasma chromosome [3]; [4]; [5]; [6] has several multi-copy genes organized in clusters of potential mobile units (PMUs), thought to be involved in host adaptation [7]. Within infected plants, phytoplasmas are restricted to phloem elements and cause growth disorders, leaf and floral alterations, and abnormal proliferation, often leading to plant death. The pathogenicity mechanisms are still unclear, but some nucleus-targeted virulence factors secreted by phytoplasma cells [8] alter plant metabolism, playing a crucial role in symptom development [9].

Phytoplasmas are transmitted by phloem-feeding insect species within the Order Hemiptera [10]. Phytoplasma transmission is persistent and propagative, involving a latent period in the vector during which ingested phytoplasmas pass from the alimentary canal through the midgut into the haemocoel, finally colonizing salivary gland cells before being transmitted to a new host plant. Vectors remain inoculative for life [11]. Phytoplasmas are usually transmitted by a narrow range of vector species [10], whereas their plant host range is usually broader [12]. Insect vector specificity plays a key role in the epidemiology of several vector-borne pathogens [13]. For this reason, identification of the molecular determinants of vector specificity is crucial to understand the epidemiology of impacting diseases worldwide. Phytoplasma colonization of the vector depends on several biological features, such as insect feeding behavior and plant host range, as well as on molecular interactions between pathogen and vector membrane proteins [11]. Since phytoplasma membrane proteins are in direct contact with host cells they are likely to be involved in specific interactions with them, as is the case for other mollicutes in which adhesion to host cells has been studied.

For example, P1 and other adhesion proteins in *Mycoplasma pneumoniae* [14]; [15], as well as other chromosomal [16] and plasmid-encoded [17]; [18]; [19] proteins in *Spiroplasma citri* are involved in interactions with host proteins.

The most abundant (immunodominant) membrane proteins of several phytoplasmas have been sequenced and classified into three types, Imp, IdpA and Amp, based on chromosomal gene organization and membrane anchor structure [20], but the specific functions of these proteins are still unknown. A strong positive selection on the Amp and Imp genes [21]; [22] is compatible with a role in host pathogen recognition. *Amp* and *Imp* are both present in the “*Ca*. P. asteris” genome, but because Amp is expressed at a higher level than Imp it is considered the predominant antigenic protein in this species [21]. A role in species-specific recognition between vector and phytoplasma has been proposed for Amp of “*Ca*. P. asteris”, onion yellows strain (OY), based on the *in vitro* interaction of OY Amp with actin of the vector and co-localization of the phytoplasma with actin filaments in the insect gut [23]. Other components of the complex phytoplasma membrane proteome also may play a role in phytoplasma-vector interactions. In ‘*Ca*. P. asteris’, plasmid-encoded transmembrane proteins are expressed to a greater degree in insects than in infected plants [24]; [7] and probably have a role in adaptation to life within the insect niche and/or in interaction of the phytoplasma membrane with the vector. The latter hypothesis is supported by the lack of an entire plasmid in a non-vector transmissible line of OY, probably due to reductive evolution [25].

Chrysanthemum yellows phytoplasma (CYP), 16SrI-B (“*Ca*. P. asteris”), is associated with a disease of ornamental plants in north-western Italy, where *Macrosteles quadripunctulatus* Kirschbaum and *Euscelidius variegatus* Kirschbaum are the most important and efficient vectors of this pathogen [26]. We have used CYP as an experimental model due to its high transmission efficiency, the rapid and clearly recognizable symptoms produced in plants and the ease of continuous insect maintenance under controlled conditions. To confirm the interaction of Amp with vector proteins, identify the insect partner molecules in the interaction and explore their role in phytoplasma transmission specificity, we used a recombinant CYP protein expressed in *Escherichia coli* [27]. Far Western blotting and affinity chromatography showed interactions of CY Amp with several proteins of the vector, among which the α and β subunits of ATP synthase were identified for the first time. No interactions with proteins of non-vector insects were observed. The presence of ATP synthase on the plasma membranes of the midgut and salivary gland cells of the insect vector was demonstrated indicating that this protein may act as a receptor for Amp. This is the first report of such a role in bacteria. Amp also interacted with actin of vector, but not non-vector, insect species, consistent with previous findings for the closely related OY phytoplasma and several of its vector species [23]. These results suggest the presence of a complex network of vector proteins that interact with Amp in the determination of vector specificity.

## Results and Discussion

All insect species used in this study were characterized for their ability to acquire and transmit CYP, for the interactions of their proteins with CYP recombinant membrane proteins (CYP antigenic membrane protein, Amp, and arginine transporter, Art) in dot far Western blots and for the capability of their proteins of binding the CYP Amp in affinity chromatography, evaluated by SDS polyacrylamide gel electrophoresis (PAGE) and Western blots of affinity column elution phases (Table 1).

**Table 1:** Insect vector status and results of interaction assays between insects and chrysanthemum yellows phytoplasma recombinant membrane proteins.

			Vectors				Non-vectors		
			Mq	Ev	Ed	St	Zp	Ac	Mp
Vector status	Acquisition		+	+^a^	+^b^	+^c^	–	+	+
	Transmission Efficiency (%)		100^a^	82^a^	2^b^	3^c^	–	–	–
Far Western blots	Amp		+	+	+	+	–	–	+
	Art		–	–	–	–	–	–	–
	His		–	–	–	–	–	–	–
	K–		–	–	–	–	–	–	–
Affinity chromatography	SDS-PAGE	p42	+	+	+	–	–	–	–
		p50	+	+	+	+/–	–	–	–
		p55	+	+	+	+/–	–	–	–
		p90	+	+	–	–	–	–	–
	WB	Actin	+	+	+	+	–	–	–
		ATP synt. β	+	+	+	+	–	–	–

a, b, c Obtained or calculated from [26], [29], [28].

Mq: *Macrosteles quadripunctulatus*; Ev: *Euscelidius variegatus*; Ed: *Empoasca decipiens*; St: *Scaphoideus titanus*; Zp: *Zyginidia pullula*; Ac: *Aphis craccivora*; Mp: *Myzus persicae*; Amp: antigenic membrane protein; Art: arginine transporter; His: histidine tag; K–: buffer devoid of protein bait; WB: Western blots.

### Vector status

*M. quadripunctulatus*, *E. variegatus*, *Empoasca decipiens* Paoli and *Scaphoideus titanus* Ball are leafhopper vectors of CYP belonging to the family Cicadellidae; the first two species are very efficient [26] while the latter two are poor vectors under experimental conditions [28]; [29]. The leafhopper *Zyginidia pullula* (Boheman), and the aphids *Aphis craccivora* Koch and *Myzus persicae* (Sulzer), putative non-vectors, were assessed for CYP transmission competence in this work; *M. quadripunctulatus* always acquired and transmitted CYP with a 100% efficiency in our experimental conditions (Table 1). *Z. pullula* (Cicadellidae) was used as a phylogenetically closely related species control; aphids were used as phloem feeder controls. All *Z. pullula* individuals (n = 11) fed on CYP infected plants were PCR-negative for the presence of the phytoplasma and, consistently, no transmission to test plants was obtained with this species. Failure of CYP transmission is explained by lack of phytoplasma acquisition by this parenchyma-feeder leafhopper [30].

Ten of 13 batches of *A. craccivora* and all 10 batches of *M. persicae* were PCR-positive after feeding on CYP infected daisy. However, none of the 10 aphid-exposed test plants for each species developed symptoms. One month after inoculation, all aphid-exposed plants tested negative in PCR for the presence of CYP. To estimate the phytoplasma titer in the aphids, PCR positive samples (10 batches each of *A. craccivora* and *M. persicae*) were analysed by quantitative real time PCR. CYP ranged between 36 and 232 cells/ng of insect DNA in *A. craccivora*, and from 256 to 1246 in *M. persicae*. Mean quantities of CYP cells per ng of insect DNA were 93 (± SE 19) and 643 (± SE 106) for *A. craccivora* and *M. persicae*, respectively. As a comparison, CYP titre in infective vector leafhoppers ranges between 2.0×10^4^ and 1.0×10^5^ phytoplasma cells/ng vector DNA, among the different species [26]. Moreover, the non transmitter individuals of the leafhopper *E. variegatus* hosted a significantly lower number of CYP cells (averaging 1800 phytoplasma cells/ng insect DNA) compared to transmitter ones [31]. Overall, pathogen titer in aphid samples was significantly lower than in infective *M. quadripunctulatus* and *E. variegatus* [26] (One way ANOVA, F = 8.927, P<0,001) and even below the amount found in non transmitter *E. variegatus* [31]. Phytoplasma acquisition by aphids is not surprising, since they feed on phloem sap, and since three species of apple aphids acquired, but did not transmit, Apple proliferation phytoplasma [32].

### Identification of interacting proteins

#### Far Western blots

**Figure 1:**
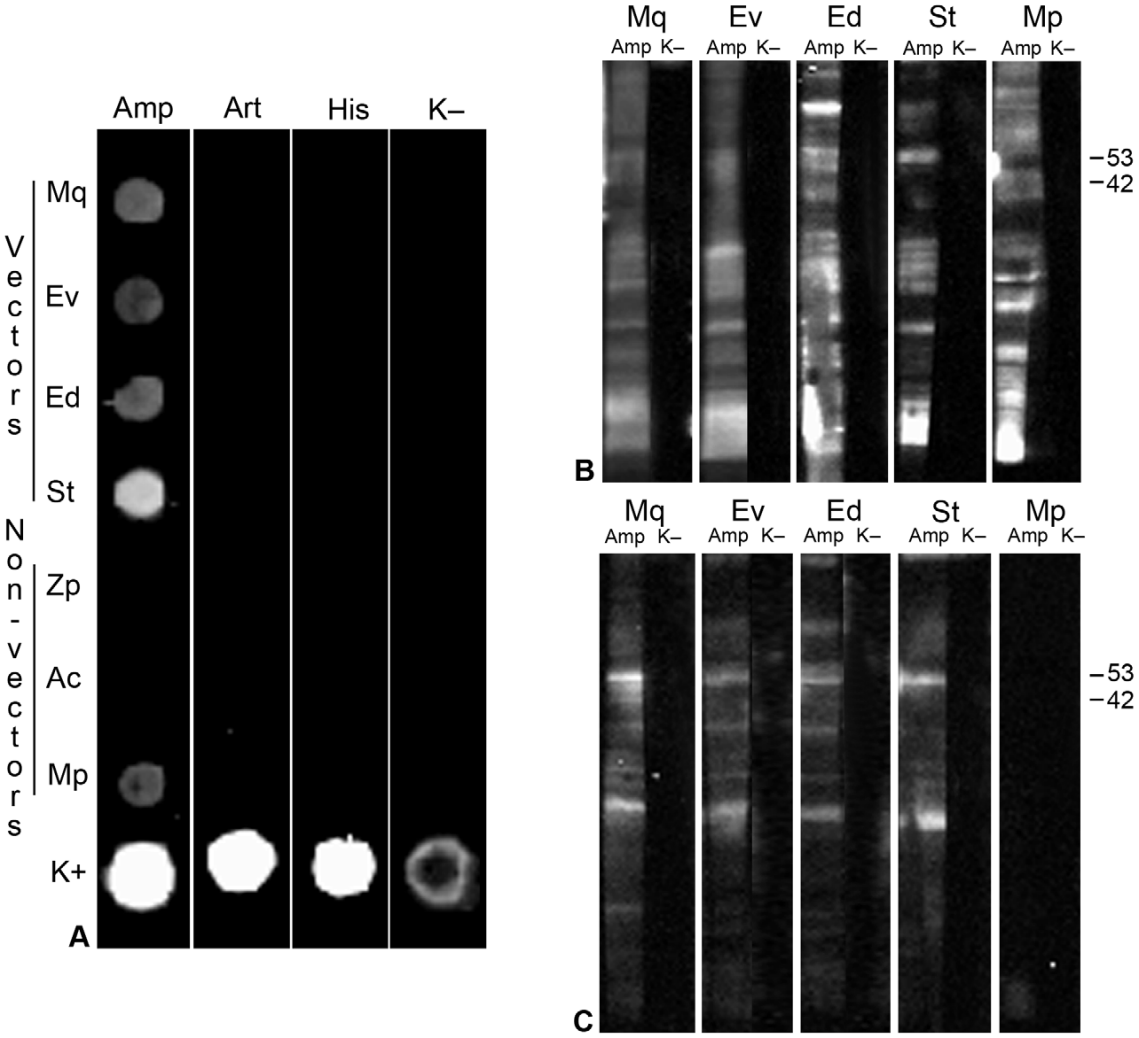
*In vitro* interaction of phytoplasma antigenic membrane protein with vector and non-vector insect proteins analysed by far Western blots. (A) Dot far Western blot: total native proteins from vector and non-vector insect species were spotted on membranes with chrysanthemum yellows phytoplasma (CYP) antigenic membrane protein (Amp) as a positive control (K+), and probed with His-tagged recombinant CYP Amp and arginine transporter (Art), histidine tag (His), and buffer devoid of protein bait (K–). Anti-HisG monoclonal antibody was used to detect bound phytoplasma recombinant proteins, and horseradish peroxidase conjugated to rabbit antimouse secondary antibody was used for chemiluminescent detection. (B, C) One dimensional far Western: binding of His-tagged recombinant CYP Amp to insect total (B) or membrane (C) proteins separated in SDS-PAGE. Secondary antibody and chemiluminescent detection were performed as in dot far Western Blot. K–: incubation in buffer devoid of protein bait. Mq: *Macrosteles quadripunctulatus*, Ev*: Euscelidius variegatus*, Ed: *Empoasca decipiens*, St: *Scaphoideus titanus*, Zp: *Zyginidia pullula*, Ac: *Aphis craccivora*, Mp: *Myzus persicae*.

To highlight any interaction between CYP fusion Amp (CYPfAmp) and insect proteins, and to observe possible differences in this interaction between vector and non-vector species, leafhopper and aphid native total proteins (serving as the prey) were spotted on a membrane and assayed in dot far Western blot (FWB) experiments, with CYPfAmp as bait (Figure 1A). Following incubation with CYPfAmp, interaction signals were detected from proteins of all vector leafhopper species (*M. quadripunctulatus*, Mq; *E. variegatus*, Ev; *E. decipiens*, Ed; *S. titanus*, St) and from the non-vector *M. persicae* (Mp), but not from the other non-vectors, *Z. pullula* (Zp) and *A. craccivora* (Ac). Incubation controls with i) the other CYP membrane-protein fusion (Art), ii) His-tag fusion antigen (His) and iii) buffer only, devoid of bait (K–), did not produce any signal (Figure 1A; Table 1). Dot blotting is a simple technique to demonstrate protein-protein interactions, but it does not provide information on the number and size of binding partners [33]. To gain clues for further identification of interacting partners, insect total proteins from vector species (Mq, Ev, Ed, St) and *M. persicae* were then separated onto one dimensional SDS-PAGE (1D FWB) and incubated with CYPfAmp (Figure 1B). Several proteins of all insect species, with molecular weights ranging between 85 and 20 kDa, interacted with CYPfAmp. In the absence of bait (K–) no interaction signal was detected. When the membrane fraction of insect proteins was assayed in 1D FWB with CYPfAmp (Figure 1C), only membrane proteins from leafhopper vector species showed interaction signals. In the absence of bait (K–) no specific interaction signal was recorded. A similar pattern of interacting membrane proteins was shared by vector species, showing intense bands of about 50 and 30 kDa.

CYP Amp always interacted with proteins of all vectors, irrespective of the insects' different transmission efficiencies [28]; [26]; [29]. No interaction with CYPfAmp was recorded with proteins of the aphid *A. craccivora* in dot FWB, whereas the interaction signal, always evident with total proteins of the aphid *M. persicae,* disappeared when membrane fractions were analysed. Therefore, we can speculate that the interacting *M. persicae* proteins may be cytosolic.

#### Affinity chromatography

**Figure 2:**
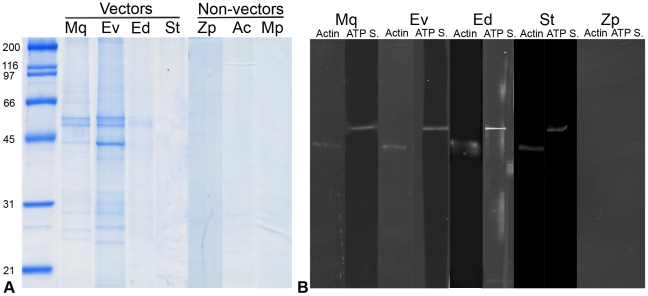
Partial purification and identification of insect vector proteins interacting with phytoplasma antigenic membrane protein. (A) Total vector and non-vector proteins, extracted under native conditions, were loaded on an affinity chromatography column covalently linked to a recombinant chrysanthemum yellows phytoplasma antigenic membrane protein. After washing, interacting insect proteins were eluted, separated by SDS-PAGE, and stained with colloidal Coomassie blue. (B) Western blots of interacting insect proteins from the affinity chromatography with antibodies against actin and ATP synthase β. Mq: *Macrosteles quadripunctulatus*, Ev*: Euscelidius variegatus*, Ed: *Empoasca decipiens*, St: *Scaphoideus titanus*, Zp: *Zyginidia pullula*, Ac: *Aphis craccivora*, Mp: *Myzus persicae*.

To identify vector proteins interacting with CYP Amp, an affinity chromatography assay was performed. Several CYPfAmp-interacting proteins from the two highly efficient vectors *M. quadripunctulatus* and *E. variegatus*, with apparent molecular weights between 25 and 90 kDa, were evident in the elution phases analysed by SDS PAGE (Figure 2A, lanes Mq and Ev). P90, p55, p50 and p42, the most abundant interacting proteins of *E. variegatus*, were excised from the gel and identified by mass spectrometry. Weak to very weak bands were present in the elution phases of the two poorly efficient vectors *E. decipiens* and *S. titanus* (Figure 2A, lanes Ed and St). In the elution phases obtained after loading the column with total extracts of non-vector species, no proteins were retained and observed in SDS-PAGE (Figure 2A, lanes Zp, Ac, Mp; Table 1). Bands were absent in SDS PAGE of elution phases following loading of vector proteins on control columns covalently linked to His-tag and CYPfArt (not shown).

Complex patterns of vector proteins interacting with CYPfAmp were obtained with FWB and affinity chromatography approaches. Similarly, several proteins interacting with Amp of the OY strain of the “*Ca*. P. asteris” have been reported for *Macrosteles striifrons* and *Hishimonus* spp. [23]. Under native conditions, CYP Amp did not interact with proteins from non-vectors (Figure 2A). Similar results were reported for OYP Amp and two non-vector leafhopper species [23].

#### Peptide mass fingerprinting and Western blots

Peptide mass fingerprinting analysis identified *E. variegatus* p55 and p50 as ATP synthase α and β subunits, respectively, and p42 as actin. Three peptides from p55 shared homology with a putative mitochondrial ATP synthase α subunit precursor of *Toxoptera citricida*, four peptides of p50 matched the ATP synthase β subunit from *Drosophila melanogaster*, and four peptides of p42 had homology with actin from *Culex pipiens pipiens* (Tables 2 and 3). Peptides from p90 did not match any known protein, and therefore p90 could not be identified.

**Table 2:** Mass spectrometry identification of proteins of the leafhopper vector *Euscelidius variegatus* interacting with chrysanthemum yellows phytoplasma antigenic membrane protein.

Interacting protein	Identified protein	Accession N.	Protein mass	Peptide count	Protein Score
p42	actin [*Culex pipiens pipiens*]	gi|90811719	41996	4	175
p50	ATP synthase β [*Drosophila melanogaster*]	gi|287945	53544	4	118
p55	ATP synthase α [*Toxoptera citricida*]	gi|52630965	59987	3	135

**Table 3:** Mass spectrometry analysis of the three proteins of the leafhopper vector *Euscelidius variegatus* interacting with chrysanthemum yellows phytoplasma antigenic membrane protein.

Protein	Observed mass	Expected Mass	Calculated mass	Peptide position^a^	Peptide sequence	Ion score
actin	945.5409	944.5336	944.5444	30–38	AVFPSIVGR	32
	1515.7296	1514.7223	1514.7419	86–96	IWHHTFYNELR	41
	1790.8822	1789.8749	1789.8846	240–255	SYELPDGQVITIGNER	46
	1486.6764	1485.6691	1485.6848	361–373	QEYDESGPGIVHR	56
ATP synthase β	1677.9087	1676.9014	1676.9210	67–81	LVLEVAQHLGENTVR	39
	1367.7260	1366.7187	1366.7456	116–127	IINVIGEPIDER	29
	1406.6729	1405.6656	1405.6739	198–211	AHGGYSVFAGVGER	18
	1988.0448	1987.0262	1987.0262	360–378	AIAELGIYPAVDPLDSTSR	31
ATP synthase α	1610.8528	1609.8455	1609.8676	132–147	TGAIVDVPVGEDLLGR	43
	1456.7858	1455.7785	1455.8409	217–229	TALAIDTIINQKR	43
	1553.7203	1552.7130	1552.7310	333–345	EAYPGDVFYLHSR	50

a Amino acid position on matching sequences (actin: gi|90811719; ATP synthase β: gi|287945; ATP synthase α: gi|52630965).

**Figure 3:**
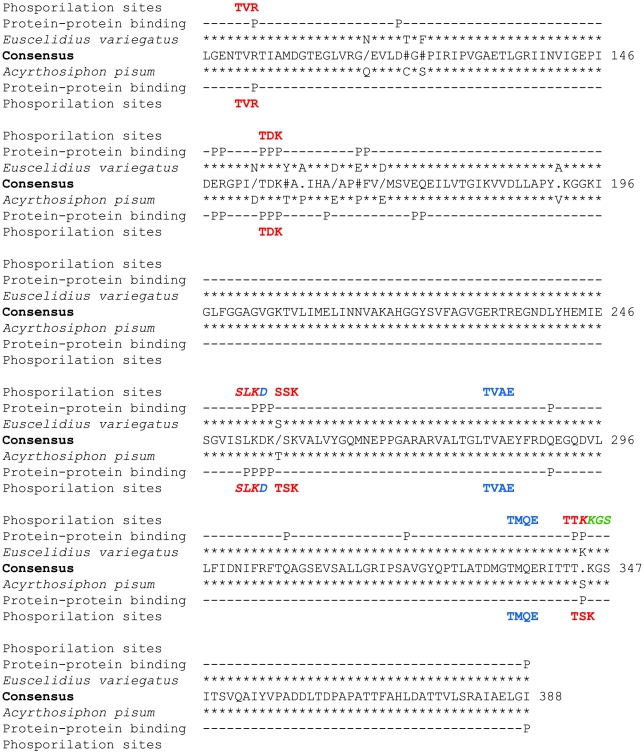
Prediction of phosphorylation and protein-protein binding sites of vector and non-vector ATP synthase β. The complete sequence of ATP synthase β subunit of the non-vector aphid *Acyrthosiphon pisum* (NP_001119645) was trimmed to the corresponding partial deduced amino acid sequence of the leafhopper vector *Euscelidius variegatus* (HQ451985), aligned with ClustalW2, and a consensus was generated and numbered on the complete *A. pisum* protein. Protein-protein binding sites (P) were predicted for each sequence (*E. variegatus* and *A. pisum*: above and below consensus line, respectively) with PredictProtein software. The same software predicted different phosphorylation sites, and these are depicted in red (protein kinase C type), blue (casein kinase II type) and green (cAMP- and cGMP-dependent protein kinase type). Overlapping phosphorylation sites are indicated in italics. *: identical amino acid;**/**: conserved amino acid substitution;.: semi-conserved amino acid substitution; **#**: non conserved amino acid substitution.

BLAST based analysis of the DNA sequences of the partial *E. variegatus* actin (HQ451984) and ATP synthase β (HQ451985) genes confirmed the MS/MS identification, and all the peptides from mass spectrometry analysis were identified in the corresponding deduced amino acid sequences. Conserved domains were found in the former sequence, confirming that the deduced protein was actin of *E. variegatus*. ClustalW alignment showed 98% similarity with the actin of the aphid *Acyrthosiphon pisum* (ACYPI006035), with one conserved and two semi-conserved substitutions. Conserved domains were found in the deduced ATP synthase β subunit protein of *E. variegatus*. ClustalW alignment showed 95% similarity with the ATP synthase β subunit of the aphid *A. pisum* (NP_001119645), with five conserved, three semi-conserved and four not conserved substitutions (Figure 3). PredictProtein analysis of the two ATP synthase β subunit sequences identified a phosphorylation site unique in the *E. variegatus* sequence, a disulphide bond site (aa 122) present only in *A. pisum*, and different profiles of protein-protein binding sites between the two insect species (Figure 3).

To further confirm protein identification, Western blot (WB) assays were carried out with actin and ATP synthase β subunit specific antibodies. The ability of these sera to detect their antigens from all the insects included in this study was preliminarly assessed: no evident difference was observed in the molecular masses of the expected proteins among the different species (data not shown).

WB analysis was performed on eluates from different phases of CYPfAmp affinity columns (Figure 2B). In the elution phases of all vectors (Mq, Ev, Ed, St), the protein p42 was recognized by the antibody against actin, and p50 by the antibody against the ATP synthase β subunit. Neither actin nor the ATP synthase β subunit were detected in the eluate of the non-vector species *Z. pullula*.

Lack of leafhopper sequence information hampered the identification of CYPfAmp-interacting p90.

Actin, and the frequently associated molecule myosin, are constitutive cytoskeleton proteins involved in several physiological processes of the cell including movement, cell shape, intracellular motility [34], phagocytosis and endocytosis [35]. Actin and myosin of leafhopper vectors interacted with Amp of the “*Ca*. P. asteris” OY strain, a phytoplasma closely related to CYP, and their role as determinants of vector specificity has been suggested [23].

Actin is a conserved gene and its interaction with Amp, a highly variable phytoplasma protein, may be necessary for conserved function. For example, actin of the vector *Circulifer haematoceps* plays a role in the internalization of *Spiroplasma citri* [16]. Involvement of host cell actin in bacterial invasion is known also for other intracellular pathogens such as *Shigella* and *Listeria* [35], *Salmonella* [36]; [37], and *Streptococcus* [38]; [39], among others. Actin is also involved in the movement of a multiple nucleopolyhedrovirus of the alfalfa looper, *Autographa californica,* towards the host nucleus during the initial phases of the infection, and towards the cell membrane tips of actin-rich surface spikes later in infection [40]. Moreover, actin and ATP synthase are both binding proteins of the CrylAc toxin found in the proteome of the midgut membrane fraction of the tobacco budworm, *Heliothis virescens* [41].

ATP synthase is an enzymatic complex responsible for ATP synthesis. Although it is generally found in the inner membrane of mitochondria [42], several recent reports describe the localization of ATP synthase components on the outer face of the plasma membrane of several human, mouse and rat cell types, where they function as receptors for multiple ligands [43]; [44] and participate in diverse processes such as regulation of lipid metabolism and cholesterol homeostasis [45]; [46], control of proliferation and differentiation of endothelial cells [47], immune recognition of tumors [48], or human innate immunity [49], as reviewed in [42] and [50]. Among arthropods, in the mosquito (*Aedes aegypti*) midgut brush border, ATP synthase β is part of a complex network of cell surface proteins, including actin, binding Dengue-2 *Flavivirus* [51]. In fat body cells of the greater wax moth, *Galleria mellonella*, ATP synthase α and β subunits have been identified in mitochondria, in the cytosol and in the plasma membrane, where they interact with juvenile hormone binding protein and have a probable role in translocation of this molecule [52]. In the crustacean *Pacifastacus leniusculus*, ATP synthase β is expressed on the membrane surface of a subpopulation of hematopoietic cells, where it acts as a receptor of a cytokine [53]. It is noteworthy that, in the Pacific white shrimp, *Litopenaeus vannamei*, ATP synthase β binds to white spot syndrome whispovirus and has a role in the host invasion process [54].

### Subcellular localization of ATP synthase

To investigate whether the β subunit of ATP synthase is exposed on the outer cell membrane of CYP insect vectors, WB assays and confocal microscopy were performed on mitochondrial protein fractions and plasma membrane.

#### SDS PAGE and Western blots

**Figure 4:**
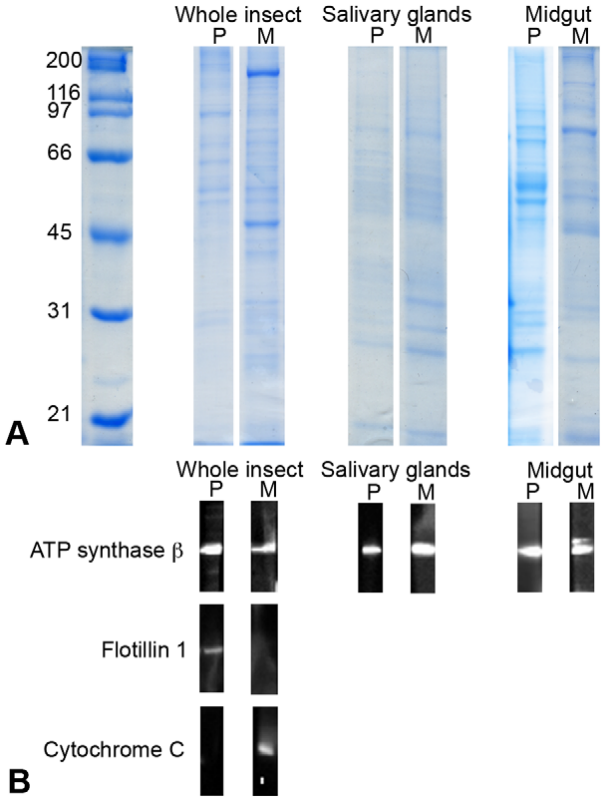
Presence of ATP synthase β in plasma membranes and mitochondrial subcellular fractions of *Euscelidius variegatus*. (A) Colloidal Coomassie blue-stained SDS-PAGE of proteins from plasma membrane (P) and mitochondrial (M) fractions from whole *E. variegatus*, or from excised salivary glands and midgut. (B) Western blots of proteins from plasma membrane (P) and mitochondrial (M) fractions of entire *E. variegatus* as well as of excised salivary glands and midgut with antibodies against ATP synthase β, flotillin 1, a plasma membrane marker, and cytochrome C, a mitochondrial marker.

Figure 4A shows SDS PAGE profiles of plasma membrane (P) and mitochondrial (M) fractions isolated from whole bodies, salivary glands and midguts of *E. variegatus*. In WB assays, flotillin 1 and cytochrome C, markers of plasma membranes and mitochondria [55], respectively, were detected only in their corresponding fraction (Figure 4B), confirming the quality of the organelle isolation protocol. ATP synthase β was detected in mitochondrial and plasma membrane fractions from whole bodies as well as from salivary glands and midgut (Figure 4B), thus confirming the expression of this protein in the plasma membranes of the two leafhopper organs most involved in phytoplasma transmission.

#### Immunofluorescence

**Figure 5:**
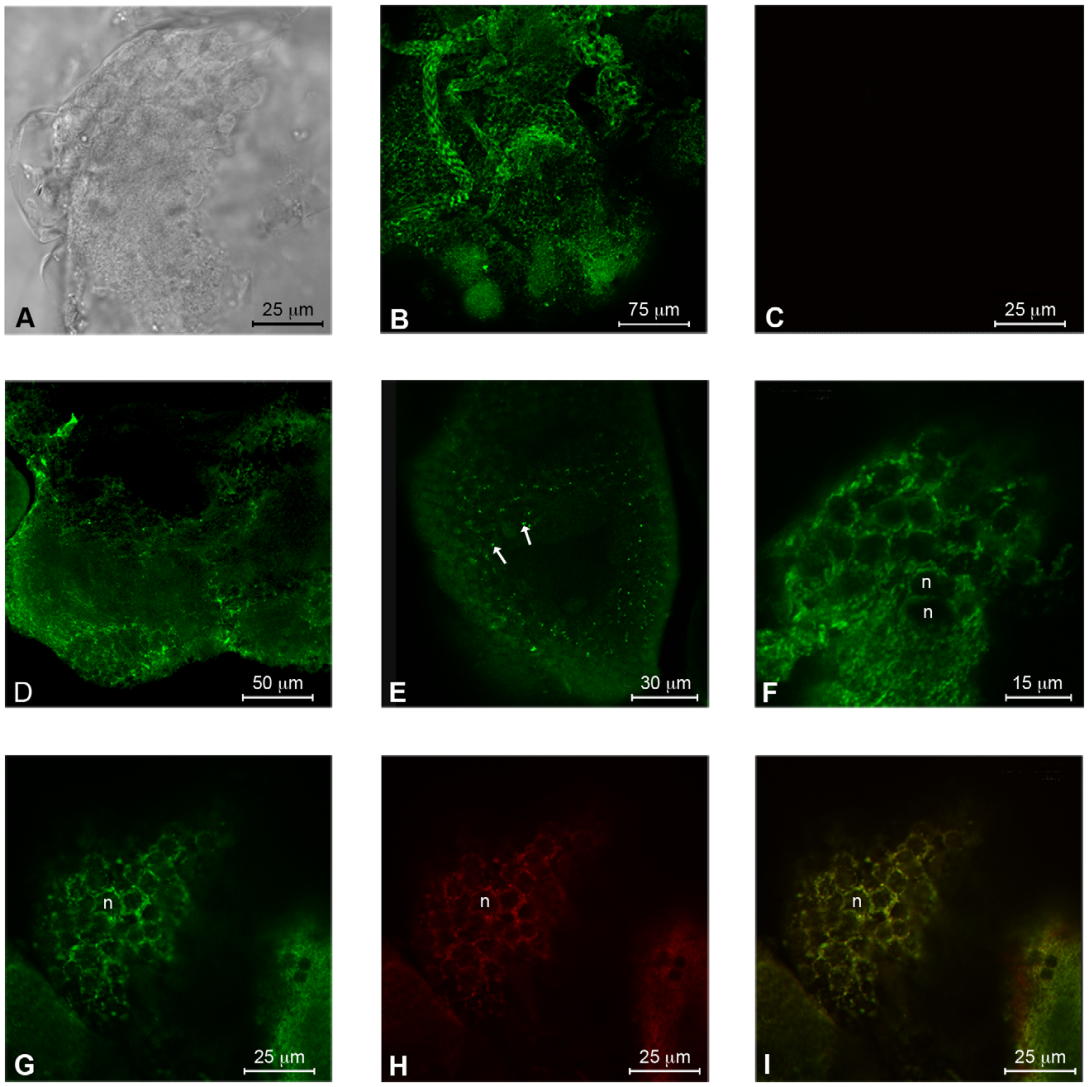
Localization of ATP synthase β on the external membranes and in the cytoplasm of salivary gland cells of the vector *Euscelidius variegatus*. Whole permeabilised salivary glands, as observed in light microscopy (A), show a strong cytoplasmic labelling of ATP synthase in immunofluorescence (B). The signal is absent in preimmune serum treatment (C). Sections of permeabilized salivary glands (D, E) also show a labelling of mitochondrial ATP synthase in the cytoplasm (arrows). In sections of not permeabilized glands (F), ATP synthase labelling is present on the cell surface. Double-labelling of ATP synthase β (G) and flotillin 1 (H) in not permeabilised sections of salivary glands reveal colocalization of the two signals, as highlighted by the resulting yellow colour in the merged image (I). n: nucleus.

**Figure 6:**
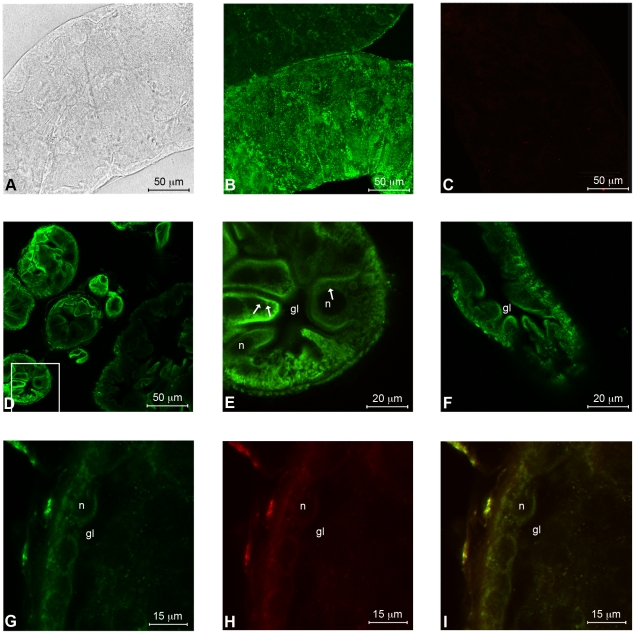
Localization of ATP synthase β on the external membranes and in the cytoplasm of midgut cells of the vector *Euscelidius variegatus*. Whole permeabilised guts, as observed in light microscopy (A), are strongly labelled by the antibody against ATP synthase β (B), while preimmune serum treated samples show no signal (C). Sections of permeabilized guts (D, E) reveal an intense labelling of mitochondrial ATP synthase β in the cytoplasm (arrows). In sections of not permeabilised guts (F), ATP synthase labelling is present on the cell surface. Double immunofluorescence labelling of both ATP synthase β (G) and flotillin 1 (H) shows the coincidence of the respective signals, resulting in the yellow colour of the merged image (I). n: nucleus; gl: gut lumen. Panel E is a magnification of panel D.

Whole permeabilised salivary glands (Figure 5B) and midguts (Figure 6B) of *E. variegatus* were strongly labelled by the anti-ATP synthase β antibody in confocal immunofluorescence microscopy. In contrast, using a rabbit pre-immune serum on similar whole organ preparations, no signal was detected (Figures 5C, 6C). Strong signals were also observed on sections of permeabilised salivary glands (Figure 5D, E) and midguts (Figure 6D, E), where mitochondria were visible as concentrated spots of signal. The anti-ATP synthase β antibody recognised the antigen also from a section of not permeabilised salivary glands (Figure 5F) and midguts (Figure 6F): in these cases the signal was most intense on cell edges, especially in the midgut, where the internal lumen brush border was strongly labelled. Finally, the same section of not permeabilised salivary glands was doubly labelled with antibodies against ATP synthase β (green, Figure 5G) and anti-flotillin 1 (red, Figure 5H), a cell membrane marker [55]: the merged image (Figure 5I) shows, in yellow, the co-localization of the two proteins. Similar observations were carried out on a section of not permeabilised midgut (Figure 6G, H, I), though the flotillin 1 signal was weaker in this organ, even in single labelling (data not shown).

The presence of ATP synthase β on the outer surface of the cell membranes of *E. variegatus* is the first demonstration of ectopic expression of this protein in the Order Hemiptera, although localization of ATP synthase was reported for plasma membrane fractions of *D. melanogaster* embryos [56].

We showed the presence of ATP synthase β in both midgut and salivary glands of a phytoplasma insect vector. These organs are essential in phytoplasma invasion of insects, the midgut being the site of hemocoel entry following feeding from the phloem of infected plants, and the salivary glands being sites of obligate colonization prior to transmission with saliva [11]. In other biological systems [57]; [58]; [43]; [59], including the freshwater shrimp [53], cell membrane ATP synthase β contributes to the production of extracellular ATP. In *S. citri*, an ATP binding cassette transporter is involved in salivary gland colonization of the vector *C. haematoceps* [60]. However, phytoplasmas lack the ATP synthetic pathway [61]; [3], and depend partly on their host for their energy metabolism [1]; [3]. Host extracellular ATP in the gut lumen and hemocoel may be required for extracellular survival of phytoplasma cells before their entrance into the midgut and salivary glands, respectively.

### Conclusion

Several lines of evidence support a role for ATP synthase as a receptor of the phytoplasma antigenic membrane protein in the transmission of CYP: i) *in vitro* interaction between Amp and insect vector proteins; ii) absence of Amp interaction with proteins of non-vector insect species; iii) localization of ATP synthase β subunit on the plasma membrane of midgut and salivary glands of the leafhopper vector *E. variegatus;* and iv) different features in the amino acid sequence of *E. variegatus* ATP synthase β compared to that of a non-vector aphid, despite high sequence similarity. Although ATP synthase activity on the surface of vector gut and salivary glands has not been investigated, extracellular ATP production in arthropods has been reported [53]. During their extracellular phase, in both the gut lumen and the hemocoel, phytoplasmas might profit from this exogenous energy source for more efficient colonization of the intestine and salivary glands, respectively. Consistent with this hypothesis, the strong interaction between CYP Amp and ATP synthase of efficient vectors correlates with high phytoplasma multiplication and transmission rates [26].

The complex profile of binding proteins retained in the affinity column in this study suggests that phytoplasma Amp interacts with a network of insect vector proteins. Among these, ATP synthase α and β were identified for the first time, and actin was confirmed in CYP as already shown in OY [23]. P90, found in this study, could not be identified, most probably due to the lack of leafhopper peptide sequence information in the databases.

We cannot exclude the existence of a highly variable host receptor among the unidentified and non conserved interacting proteins. In this case, the interaction of CYP Amp with the highly conserved proteins ATP synthase, actin, and myosin could be mediated by such a receptor. Nevertheless, the freeze-thawing disruption of putative insect protein complexes before affinity chromatography did not alter the profile of Amp-interacting proteins (data not shown), suggesting that Amp interaction with actin and ATP synthase α and β could be direct. Moreover, the phytoplasma membrane proteome is complex, including several transport systems [3] and at least two antigenic membrane proteins [21], and it may also be dynamic, as suggested by the recent report of membrane-targeted proteins expressed differentially in the vector and encoded by extrachromosomal potential mobile units [7]. Recently, a role in phytoplasma transmission has been suggested also for a transmembrane plasmid-encoded protein of OY [24]. The role of other phytoplasma membrane proteins in interactions with insect proteins must be further explored to fully understand the mechanism of phytoplasma transmission by vector insects.

## Materials and Methods

### Phytoplasma strain, insect species and transmission experiments

Chrysanthemum yellows phytoplasma (CYP) [26] was maintained in daisy, *Chrysanthemum carinatum* Schousboe, by periodic insect transmission using *M. quadripunctulatus.* CYP vectors *M. quadripunctulatus* and *E. variegatus* were reared on oat (*Avena sativa* (L.)), *E. decipiens* on broad bean (*Vicia faba* (L.)), and *S. titanus* on grapevine (*Vitis vinifera* (L.)). *Z. pullula* was reared on corn (*Zea mays* (L.)), *A. craccivora* on broad bean, and *M. persicae* on pepper (*Capsicum annuum* (L.)). All insect species were collected in the Piemonte region of Italy. For transmission experiments, about 100 nymphs (4th-5th instar) of *Z. pullula* were confined on CYP-infected daisy (PCR positive) for three days and then maintained on corn for three weeks (latent period), under climatic chamber conditions (T = 25°C, L:D = 16h:8h). Surviving adults were transferred for an inoculation access period of three days onto five daisy test plants within one cage to check CY transmission ability, collected and checked by nested PCR for the presence of CY phytoplasma. Aphids (about 500) were confined on CYP-infected daisies for three days and then maintained on healthy broad bean or pepper plants for four days. Some insects were then assayed in batches of 10 by nested PCR, whereas others (at least 200) were confined for life on 10 daisy test plants to check CY transmission ability. Nymphs of *M. quadripunctulatus* were always caged together with *Z. pullula*, *A. craccivora* and *M. persicae* on infected source plants (PCR positive) as acquisition controls and separately checked for CYP transmission to healthy daisies. Visual symptom assessment and PCR assays of total DNA from leaf veins were used to assess phytoplasma infection of the exposed plants.

### DNA extraction, diagnostic nested PCR and quantitative real time PCR

Total DNA was extracted from leaf veins of exposed plants, from individual *Z. pullula* adults and from batches of 5/10 aphids, as described [62]. The presence of CYP was assayed by nested PCR driven with universal and specific primers [63]; [64]. The concentration of total DNA in PCR positive insects was measured using quantitative real time PCR, as described in [62]. CYP DNA was expressed as number of CYP cells per nanogram of insect DNA. To compare phytoplasma titre in aphids and in infective *M. quadripunctulatus* and *E. variegatus* [26], one-way analysis of variance (ANOVA) followed by Tukey test for multiple comparison was performed with SIGMAPLOT 11 (Systat Software).

### Protein extraction

Total proteins were extracted from batches of all insect species (20 *M. quadripunctulatus*, 10 *E. variegatus*, 25 *E. decipiens*, 10 *S. titanus*, 30 *Z. pullula* and about 100 individuals of each aphid species) with the same procedure to perform dot far Western blots (FWB), one dimensional (1D) FWB, Western blots (WB) and affinity chromatography experiments. Insects were homogenized with a mortar and pestle in 200 µl of Rx buffer [23], and centrifuged for 1 min at 13,000 g; proteins in the supernatant were quantified with Bradford reagents (Bio-Rad).

For 1D FWB membrane proteins were extracted, according to a protocol adapted from [65]. Insects were crushed in 200 µl of Buffer 1 (1% Triton X-114, 150 mM NaCl, 10 mM Tris, pH 7.4), sonicated for 1 min and centrifuged for 3 min at 13,000 g at 0°C. The supernatant was layered onto 300 µl of a sucrose cushion (6% sucrose, 0.06% Triton X-114, 150 mM NaCl, 10 mM Tris, pH 7.4), incubated 3 min at 30°C and centrifuged at room temperature (RT) at 300 g for 3 min. The top (aqueous) phase, about 200 µl, was collected, mixed with 300 µl of fresh Buffer 1 and incubated on ice for 3 min; the bottom (detergent) phase kept at RT. The aqueous phase was loaded, for a second separation, on the same detergent phase. Following an incubation of 3 min at 30°C and centrifugation at RT (300 g for 3 min), the detergent phase containing membrane proteins was collected (∼30 µl) and the protein concentration was estimated using the Bradford assay, as before.

### Subcellular fractionation

To determine the cellular localization of ATP synthase, mitochondria and plasma membranes were separated from whole bodies or excised salivary glands and midguts of *E. variegatus*, as described [57]. Whole insects (50 specimens) were crushed in 600 µl of 9% TES buffer (20 mM Tris HCl, 1 mM EDTA, 9% sucrose, pH 7.4), and centrifuged at 500 g for 5 min at 4°C. The supernatant was homogenized in 9% TES buffer in a Potter-Elvehjem homogenizer and centrifuged at 14,0000 g for 30 min at 4°C. The pellet was resuspended in 1 ml of 9% TES buffer layered on a cushion of 1 ml of TES buffer containing 50% sucrose and 1 ml of TES containing 38.5% sucrose, and centrifuged at 100,000 g in a TLA-100.3 rotor (Beckman-Coulter) for 60 min at 4°C. Plasma membranes were collected from the top of the sucrose cushion, suspended in 9% TES buffer and pelletted by centrifugation at 31,000 g for 60 min at 4°C. Mitochondria were collected from the bottom of the sucrose cushion, suspended in 9% TES buffer and pelletted by centrifugation at 5,000 g for 60 min at 4°C. Excised salivary glands and midguts from 30 and 100 *E. variegatus*, respectively, were homogenized in 9% TES buffer in a Potter-Elvehjem homogenizer and treated as described above.

### Far Western blots

Partial fusion proteins of CYP, Amp (Antigenic membrane protein) and Art (Arginine transporter), produced previously [27], were used as bait in FWB experiments, following a protocol slightly modified from [66]. For dot FWB, 40 µg total proteins of each insect species was spotted on polyvinyl difluoride (PVDF, Bio-Rad) membrane; 1 ng of CYPfAmp was also spotted, as positive control. For 1D FWB, insect total or membrane protein preparations were subjected to SDS-PAGE (40 µg/well) and blotted onto PVDF membrane. Dot FWB and 1D FWB membranes were blocked for 1 h at 4°C with 3% BSA in TBS containing 0.1% Tween (TBST) and then incubated overnight at 4°C with bait recombinant proteins: i) CYPfAmp, ii) CYPfArt, iii) His-tag purified from pRSetC expression vector (Invitrogen), or iv) buffer only (negative control). Four µg of all baits were suspended in BSA-TBST mixed with EDTA-free antiprotease cocktail Complete I (Roche). The PVDF membranes were washed for 1 h with BSA-TBST and for 10 min with TBST, then incubated 4 h with anti-HisG monoclonal antibody (R940-25, Invitrogen), washed 3 times in BSA-TBST, incubated 2 h with horseradish peroxidase (HRP) conjugated rabbit antimouse secondary antibody (A0545, Sigma-Aldrich), and washed three times with TBST. Primary and secondary antibodies were diluted to 1∶5000 in BSA-TBST. For all experiments, detection was performed with West Pico SuperSignal chemiluminescent substrate (Pierce) and a VersaDoc 4000 MP (Bio-Rad). Each experiment was repeated at least three times, each time with freshly extracted insect proteins.

### Western blots

Western blots (WB) were carried out to confirm the identification of Amp interacting insect proteins, using antisera specific for actin and for ATP synthase β. This technique also was used to analyse the subcellular localization of ATP synthase; in addition to the anti-ATP synthase β antibody, an antiserum specific for flotillin 1, a protein expressed only in the plasmalemma and used as a cell membrane marker, and another antiserum specific for cytochrome C, a mitochondrial marker [55], also were used. Following SDS-PAGE on 12-15% polyacrylamide gels, proteins were blotted on PVDF membrane. Membranes were blocked, incubated with primary and secondary antibodies diluted in BSA-TBST, washed and developed as detailed above for the FWB assays. Polyclonal primary antibodies, specific for ATP synthase β (ab43177, Abcam plc, diluted 1∶5000), actin (A5060, Sigma-Aldrich, diluted 1∶1000) and flotillin 1 (ab41927, Abcam plc, diluted 1∶1000), were detected with HRP conjugated antibody A0545 (Sigma-Aldrich, diluted 1∶10,000). The monoclonal anti-cytochrome C (ab13575, Abcam plc, diluted 1∶1000) was recognized by HRP conjugated A4416 (Sigma-Aldrich, diluted 1∶5000).

### Affinity chromatography and protein identification

One mg of total proteins from different insect species was loaded on an affinity column (AminoLink Kit, Pierce) covalently linked to 2 mg of CYPfAmp, according to the manufacturer's instructions. Six fractions of about 1.5 ml were collected from each elution. Proteins in each fraction were precipitated with acetone, subjected to SDS-PAGE and either colloidal Coomassie blue stained or blotted on PVDF membrane for WB. Affinity chromatography assays were repeated at least three times for each insect species, always with freshly extracted proteins.

Four visible bands in the elution phases of *E. variegatus* were excised from the gel and shipped to the Protein ID Service (Department of Biology Technology Facility, University of York, UK) for trypsin digestion, and MALDI-MS and MS/MS analysis with a Bruker Autoflex III MALDI-TOF/TOF. Protein identification was accomplished using the MASCOT database search engine (www.matrixscience.com).

### Primer design, RT-PCR, and sequence analysis

To further confirm the MS/MS protein identification we obtained the corresponding DNA coding sequences. Actin and ATP synthase β genes from several insect species, available in Genbank, were aligned with ClustalW2 [67] and degenerated primers were designed on consensus DNA fragment sequences encoding peptides identified by mass spectrometry of *E. variegatus* proteins. Selected primers were ActFw1 (5′-ATGTGTGACGAWGAKRTWGCMGC-3′) ActRv1100 (5′-CCDGGGCCGGAYTCGTCGT-3′) for actin, and BetaFw220 (5′-CAGCAYTTGGGWGAAAAYAC-3′) BetaRv1110 (5′-GGATCBACAGCWGGRTARATACC-3′) for ATP synthase β. Total RNA was extracted from five *E. variegatus* adults with TRIzol® reagent (Invitrogen) and reverse transcribed to cDNA with MuLV® Reverse Transcriptase and Random Hexamers (Applied Biosystems) following manufacturer's recommendations. *E. variegatus* cDNA was amplified with degenerated primers and PCR products, corresponding to actin and ATP synthase β partial genes, were sequenced with a capillary 3730 DNA Analyzer (ABI) by Bio-Fab Research (Pomezia, RM, Italy). Programs ClustalW2 [67], Conserved Domain Database [68] and PredictProtein [69] were used to analyse sequence features.

### Immunofluorescence

Salivary glands and midguts were excised from *E. variegatus* adults and fixed quickly in 4% paraformaldehyde, 0.1 M phosphate buffer, pH 7.4, 0.1% Triton X-100 overnight at 4°C. Organs were washed three times in phosphate-buffered saline, pH 7.4 (PBS) and some were permeabilised overnight at 4°C with PBS and 1% Triton X-100. Confocal microscopy was performed on whole organs and on 100 µm thick sections, made with a Balzer Vibratome series 1.000 from 8% agarose embedded organs. Whole organs or sections were blocked in PBS containing 1% BSA for 30 min, incubated overnight with anti-ATP synthase β antibody (ab43177, Abcam plc, diluted 1∶200), washed three times with PBS, blocked, incubated for 2 h with a 1∶80 dilution of the FITC conjugated antibody (F1262, Sigma-Aldrich), and finally washed five times in PBS. Samples were mounted on microscope slides in Citifluor (Molecular Probes, Invitrogen) and observed. Control sections were treated in a similar manner but incubated in a rabbit pre-immune serum (diluted 1∶200) instead of primary antibody. A sequential labelling protocol was employed to double label ATP synthase and flotillin 1 and confirm the ectopic expression of the former. We first labelled ATP synthase with FITC as described above. Sections were then incubated overnight with anti-flotillin 1 antibody (ab41927, Abcam plc) diluted 1∶150, and 3 h with Alexa 633 conjugated antibody (A-21071, Molecular Probes, Invitrogen) diluted 1∶50. The control sections were treated in a similar manner, except that either the second or both antibodies were omitted from the incubation mixture. All samples were observed with a Leica TCS SP2 confocal microscope, using a 40× water-immersion objective (HCX Apo 0.80). Laser bands of 488 nm Ar and a 633 nm He/Ne were used to excite FITC and Alexa 633, respectively.
